# Digital self-management of hip and knee osteoarthritis and trajectories of work and activity impairments

**DOI:** 10.1186/s12891-023-06322-z

**Published:** 2023-03-18

**Authors:** Ali Kiadaliri, L. Stefan Lohmander, Majda Misini Ignjatovic, Håkan Nero, Leif E. Dahlberg

**Affiliations:** 1 Department of Clinical Sciences Lund, Orthopaedics, Clinical Epidemiology Unit, Lund University, Lund, Sweden; 2 Centre for Economic Demography, Lund University, Lund, Sweden; 3 Arthro Therapeutics, Malmö, Sweden; 4 Clinical Epidemiology Unit, Skåne University Hospital, Remissgatan 4, Lund, SE-221 85 Sweden; 5 Department of Clinical Sciences Lund, Orthopaedics, Lund University, Lund, Sweden

**Keywords:** Activity impairment, Digital therapy, Longitudinal trajectory, Osteoarthritis, Self-management, Sweden, Work impairment

## Abstract

**Objective:**

To investigate the trajectories of work and activity impairments among people participating in a digital self-management program for osteoarthritis (OA).

**Methods:**

We conducted an observational longitudinal study using data for baseline, 3, 6, 9 and 12 months follow ups from people participating in a digital OA treatment between June 2018 and September 2021. The Work Productivity and Activity Impairment–Osteoarthritis (WPAI–OA) questionnaire was used to measure work and activity impairments. We applied linear mixed models and group-based trajectory modelling (GBTM) to assess the trajectories of work and activity impairments and their variability. Dominance analysis was performed to explore the relative importance of baseline characteristics in predicting the trajectory subgroup membership.

**Results:**

A total of 14,676 participants with mean (± standard deviation) age 64.0 (± 9.1) years and 75.5% females were included. The adjusted mean improvements in work impairment from baseline were 5.8% (95% CI 5.3, 6.4) to 6.1% (95% CI 5.5, 6.8). The corresponding figures for activity impairment were 9.4% (95% CI 9.0, 9.7) to 11.3% (95% CI 10.8, 11.8). GBTM identified five (low baseline–declining, moderate baseline–declining, high baseline–declining, very high baseline–substantially declining, and very high baseline–persistent) and three (low baseline–declining, mild baseline–declining, high baseline–declining) subgroups with distinct trajectories of activity and work impairments. Dominance analysis showed that baseline pain was the most important predictor of membership in trajectory subgroups.

**Conclusion:**

While participation in a digital self-management program for OA was, on average, associated with improvements in work and activity impairments, there were substantial variations among the participants. Baseline pain may provide useful insights to predict trajectories of work and activity impairments.

**Supplementary Information:**

The online version contains supplementary material available at 10.1186/s12891-023-06322-z.

## Introduction

Osteoarthritis (OA), the most common form of arthritis, is a steadily growing disease associated with pain, disability, and deteriorated quality of life [1–3]. Although there is no cure for OA, exercise and education are recommended as first-line core treatments [4]. To promote delivery and uptake of these treatments, self-management programmes have been initiated in different countries [5]. Despite effectiveness of these programs [6], they have mostly been implemented at small-scale, leaving many patients with limited access to instruction and/or motivation for self-management [7, 8]. Hence, cost-effective interventions that are accessible to most patients are needed to help them adopting and maintaining long-term self-management.

In response to this and in light of the explosion of smart technology as well as restrictions imposed by the COVID-19 pandemic, there has been a remarkable rise in popularity of digital therapeutics including smartphone apps in OA management over recent years [9]. Studies have reported improvements in pain and physical function following digital self-management interventions compared to usual care in people with OA [9, 10]. In addition to pain and physical function, OA influences work productivity and daily activities substantially [11, 12]. Indeed, productivity losses might comprise a larger proportion of total costs attributable to OA compared with healthcare expenses [13, 14]. Despite this, little is known about the effects of digital self-management interventions on work productivity and activity impairments. The main aim of present study was to investigate the changes in work and activity impairments up to 1 year following participation in a digital self-management program for OA in Sweden. We also explored potential heterogeneity in these changes in our sample.

## Methods

### Study design and data source

This was an observational longitudinal study on participants of Joint Academy® (JA), a digital self-management program for OA [15]. All data were collected through participants’ use of the app. Participants in the digital program either had a prior radiographic and/or clinical diagnosis of hip or knee OA from a physical therapist or physician or were confirmed to have clinical OA by physiotherapist via phone [16].

### Digital self-management program

JA was introduced in Sweden in 2016 as a digital version of the evidence-based structured first-line face-to-face self-management program, known as “Better management of patients with OsteoArthritis (BOA)” [17]. The digital program has been described in detail [15, 16]. In brief, it comprises video lectures on OA, physical activity, and self-management (a total of 70 lectures over a 48-week period) as well as individualised neuromuscular exercises with complexity and difficulty level that are adjusted to each participant’s progression in the program. The participants are supervised during the full participation period and have an option to chat asynchronously with an assigned physical therapist for feedback and questions [15, 16]. The program is available and reimbursed via the national health-care system for all patients in Sweden.

### Participants

**Fig. 1:**
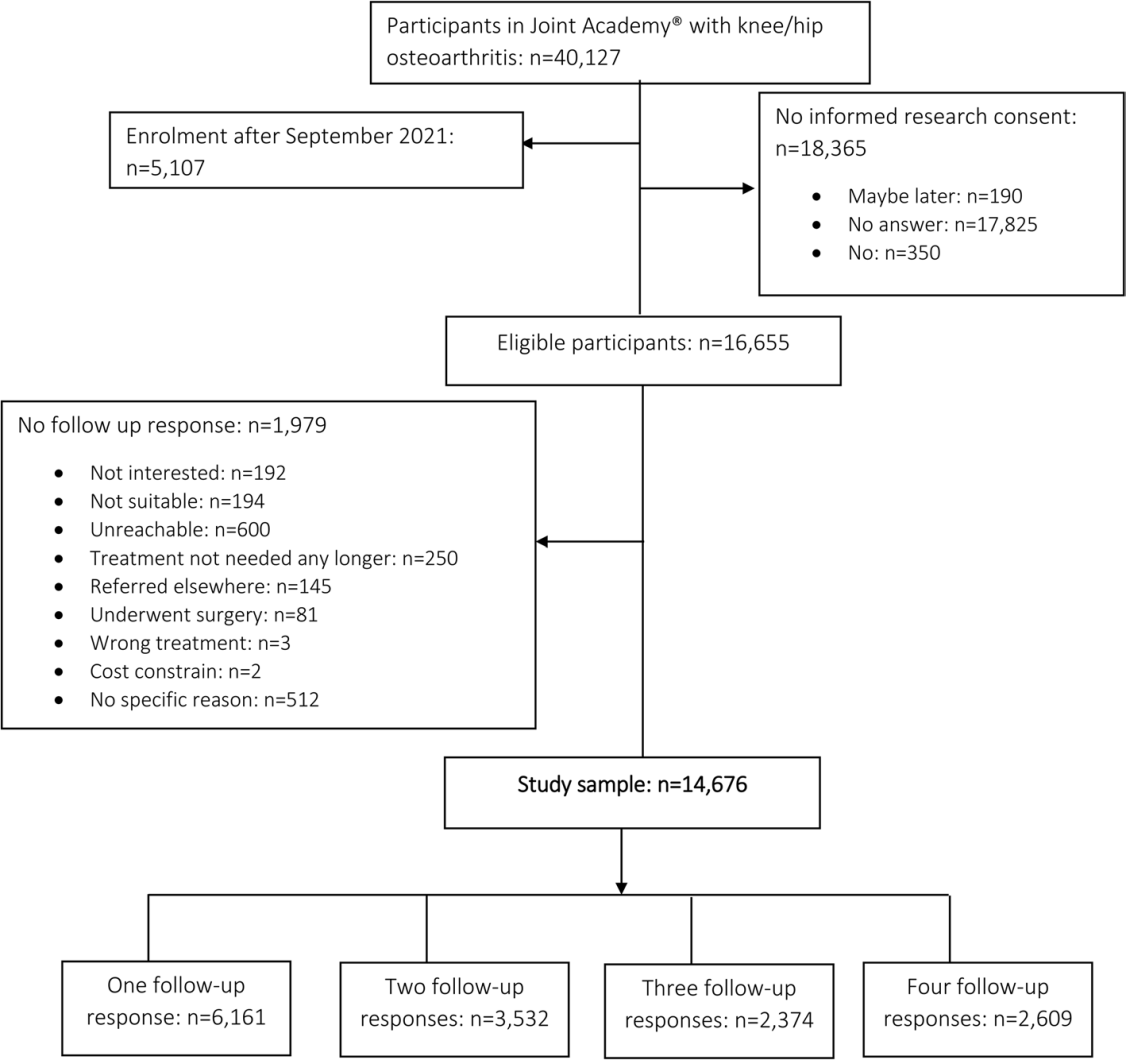
Flow chart of the study population

Data were retrieved on January 14, 2022 from consecutive participants with hip or knee OA enrolled in the digital program between June 2018 and September 2021 (*n* = 35,020, Fig. 1). Participants joined the digital program through recommendation by their local orthopaedic surgeon or physiotherapist, or via online advertisements and campaigns placed on search engines and social networks. Of these, we excluded 18,365 (52.4%) individuals who didn’t provide their informed consent for research, as well as 1,979 (5.7%) persons with no follow up responses. These exclusion criteria resulted in a final sample of 14,676 individuals included in this study. Among these, 15 (0.1%) persons were enrolled in the year 2018, 933 (6.4%) in 2019, 3,697 (25.2%) in 2020, and 10,031 (68.4%) in 2021.

### Measure

The Work Productivity and Activity Impairment–Osteoarthritis (WPAI–OA) questionnaire was used to measure work and activity impairments [18]. WPAI has been suggested as the preferred patient measure for measuring work productivity among persons with rheumatic diseases [19] with an intraclass correlation coefficient (ICC) of 0.74 [20]. WPAI is a six-item validated instrument covering four metrics: “absenteeism”, “presenteeism”, “overall work impairment” (absenteeism + presenteeism), and “activity impairment” [18]. Absenteeism measures the percent work time missed due to OA and is calculated as [hours missed due to OA/ (hours missed due to OA + hours actually worked)]. Presenteeism measures the extent to which OA affected productivity while working. This was estimated by multiplying the percent actually working by the extent of work impairment due to OA. The work impairment due to OA was measured using a 11-point numerical rating scale (NRS, 0 = OA had no effect on my work and 10 = OA completely prevented me from working) expressed as percentage by multiplying by 10. Overall work impairment is calculated as: absenteeism + presenteeism. Activity impairment measures the extent to which OA influenced the ability to do regular daily activities using a 11-point NRS (0 = OA had no effect on my daily activities and 10 = OA completely prevented me from doing my daily activities) and is expressed as percentage by multiplying by 10. All these metrics are expressed as impairment percentages with higher numbers indicating worse outcomes, i.e. greater impairment and less productivity [18]. WPAI has a 7-day recall period (i.e., absenteeism, presenteeism and activity impairment during the past 7 days). Data were collected at baseline and months 3, 6, 9, and 12 following participation in the program.

### Covariates

The following explanatory variables measured at baseline were included in our analysis: sex, age, body mass index (BMI), index joint (knee or hip), education, employment status, specific self-reported coexisting conditions (diabetes, lung diseases, balance troubles, rheumatoid arthritis, cardiovascular diseases, and walking difficulties), pain, general health, and physical function. Pain was assessed using an 11-point NRS (0 = no pain and 10 = the worst possible pain). General health was measured using an 11-point NRS (“Mark on the scale how good or bad your current health is?” with 0 = worst imaginable and 10 = best imaginable). Physical function was measured using the 30-s chair stand test (30CST) in which the participants sit and stand from a chair for 30 s and reported the maximum number of repetitions.

### Data analysis

To investigate the average trajectory in work and activity impairments, we applied a linear mixed effect regression model with individuals as random effect and follow up time (as categorical variable) as fixed effect (random intercept model with “identity” covariance structure and robust standard errors). The regression model was adjusted for the baseline values of the covariates (described above) as well as the baseline value of the outcome variable of interest (i.e. work impairment or activity impairment). In subgroup analyses, we investigated the potential differences in the average trajectories by sex, age, and index joint. This was implemented by inclusion of the interaction between these variables with time in the regression models. The results are presented as predicted marginal means with 95% confidence intervals (CI) using the Stata program’s “margins” command. Two separate sensitivity analyses excluding those with adherence < 80% at 3-month follow up and excluding those with a missing follow up response (complete case analysis) were conducted. The residual diagnostics confirmed that the models’ assumptions were fulfilled. Work impairment was studied only among the participants aged 65 years and younger who were employed.

To capture potential variability in trajectories of work and activity impairments, we applied semi-parametric group-based trajectory modelling (GBTM) [21]. GBTM is a data-driven technique dividing the participants into classes assuming those within each class follow exactly the same trajectory of an outcome over time [21]. We used censored normal distribution in our estimation. First, we estimated models with 2 to 7 latent classes with cubic polynomials and selected the optimal number of classes based on following criteria: The Bayesian Information Criterion (BIC, lower value indicates better fit), average posterior probability of class membership (> 0.7 for each class), the odds of correct classification (> 5 for each class), class size (≥ 5% of participants in the smallest class), and relative entropy (values closer to 1 reflect better fit), and model parsimony and interpretability [21, 22]. After selecting the number of classes, we estimated models with all possible combinations of constant, linear, quadratic, and cubic polynomials and selected the final model based on the criteria listed above. After estimating the final model, we used the posterior probability to assign each individual to the class with the highest probability. GBTM was implemented using Stata’s “traj” command [23]. We then used class membership as an outcome and explored its associations with the baseline characteristics using multinomial logistic regression. To explore the relative importance of each predictor in predicting class membership, we computed the contribution of each predictor to the change in the McFadden pseudo-*R*^2^ from multinomial logistic regression across all possible subset models using dominance analysis (using Stata’s “domin” command) [24]. We also assessed the predictive accuracy of the baseline characteristics in predicting trajectory class membership using the polytomous discrimination index (PDI), Matthews correlation coefficient (MCC) and the confusion entropy (CEN) [25]. For an outcome with k categories, PDI is the probability of correctly identifying a subject selected randomly from a random sample of k subjects (one subject from each category) with random accuracy equal to 1/k (with k = 2, PDI is equivalent to the standard c-statistics) [26]. MCC ranges from -1 (worst predictive accuracy) to + 1 (perfect predictive accuracy) and values close to 0 indicating random accuracy. CEN ranges from 0 (perfect accuracy) to 1 (worst accuracy). PDI was computed using “pdifunction” in R [26] and MCC/CEN were computed using “PyCM” package in Python [27].

## Results

A total of 14,676 participants with mean (± standard deviation) age 64.0 (± 9.1) years and 75.5% females were included (Table 1). Compared to the participants included in the study, those excluded (*n* = 1979) were, on average, older with higher proportions of co-existing conditions and slightly greater work and activity impairments at baseline. Among participants included in the study, 4,983 (34.0%) participants had at least three follow up responses in addition to their baseline response. This proportion was 59.0% among persons enrolled prior to the year 2021 and 22.5% among those enrolled in the year 2021 (this was expected given that the data was extracted in January 2022). A larger proportion (60.0%) of the participants reported the knee as their most painful (index) joint. Most participants (80.5%) were 51–74 years old and had a college/university degree (56.0%). Walking difficulties followed by lung diseases and cardiovascular diseases were the most common coexisting conditions. There were, on average, high levels of adherence to the treatment among participants (86.3% at 3-month to 84.1% at 12-month follow ups). Among participants aged 65 years and younger who were employed at baseline (*n* = 5,186), 93.4% and 32.1% reported no absenteeism and presenteeism, respectively, during last 7 days. Proportion of participants with no activity impairment at baseline was 7.5% in the sample. There was, on average, mild work impairment (24.3%, 95% CI: 23.6, 24.9) mainly due to presenteeism and moderate activity impairment (39.3%, 95% CI: 38.9, 39.7) at baseline.

**Table 1:** Baseline characteristics of the participants

	Included			Excluded
	Knee (*n* = 8,801)	Hip (*n* = 5,875)	Total (*n* = 14,676)	*n* = 1,979
Female, n (%)	6,530 (74.2)	4,549 (77.4)	11,079 (75.5)	1493 (75.4)
Age, mean (± SD)	64.0 (± 9.0)	64.1 (± 9.2)	64.0 (± 9.1)	65.7 (± 10.3)
21–50 years, n (%)	648 (7.4)	443 (7.5)	1,091 (7.4)	158 (8.0)
51–65 years, n (%)	4,184 (47.5)	2,691 (45.8)	6,875 (46.9)	755 (38.2)
66–74 years, n (%)	2,931 (33.3)	2,002 (34.1)	4,933 (33.6)	678 (34.3)
75 + years, n (%)	1,038 (11.8)	739 (12.6)	1,777 (12.1)	388 (19.6)
Education, n (%)				
Less than high school	701 (8.0)	483 (8.2)	1,184 (8.1)	180 (9.1)
High school	3,149 (35.8)	2,120 (36.1)	5,269 (33.9)	703 (35.5)
College/university	4,951 (56.3)	3,272 (55.7)	8,223 (56.0)	1,096 (55.4)
Body mass index, mean (± SD)	27.6 (± 4.9)	26.5 (± 4.4)	27.2 (± 4.7)	27.2 (± 4.8)
Employment, n (%)				
Working	3,944 (44.8)	2,537 (43.2)	6,481 (44.2)	707 (35.7)
Not working	445 (5.1)	272 (4.6)	717 (4.9)	139 (7.0)
Retired	4,412 (50.1)	3,066 (52.2)	7,478 (51.0)	1,133 (57.3)
Diabetes, n (%)	504 (5.7)	318 (5.4)	822 (5.6)	171 (8.6)
Lung diseases, n (%)	917 (10.4)	642 (10.9)	1,559 (10.9)	227 (11.5)
Balance troubles, n (%)	284 (3.2)	205 (3.5)	489 (3.3)	112 (5.7)
Rheumatoid arthritis, n (%)	396 (4.5)	273 (4.7)	669 (4.6)	130 (6.6)
Cardiovascular diseases, n (%)	622 (7.1)	462 (7.9)	1,084 (7.4)	211 (10.7)
Walking difficulties, n (%)	1,027 (11.7)	779 (13.3)	1,806 (12.3)	317 (16.0)
General health, mean (± SD)	6.6 (± 1.9)	6.5 (± 1.8)	6.6 (± 1.8)	6.4 (± 2.0)
Pain, mean (± SD)	5.1 (± 2.0)	5.1 (± 1.9)	5.1 (± 1.9)	5.2 (± 2.1)
Physical function, mean (± SD)	12.6 (± 4.3)	13.0 (± 4.4)	12.8 (± 4.3)	12.0 (± 4.4)
WPAI–OA activity impairment (%), mean (± SD)	40.3 (± 23.8)	37.9 (± 23.4)	39.3 (± 23.7)	41.8 (± 24.9)
WPAI–OA absenteeism (%), mean (± SD)^a^	2.7 (± 13.4)	2.1 (± 11.4)	2.4 (± 12.7)	2.8 (± 13.0)
WPAI–OA presenteeism (%),mean (± SD)^a^	21.7 (± 22.8)	22.0 (± 21.2)	21.8 (± 22.2)	23.2 (± 22.7)
WPAI–OA overall work impairment (%), mean (± SD)^a^	24.4 (± 25.7)	24.1 (± 23.7)	24.3 (± 24.9)	25.9 (± 25.6)
Follow up responses, n (%)				
1	3,614 (41.1)	2,547 (43.4)	6,161 (42.0)	-
2	2,087 (23.7)	1,445 (24.6)	3,532 (24.1)	-
3	1,481 (16.8)	893 (15.2)	2,374 (16.2)	-
4	1,619 (18.4)	990 (16.9)	2,609 (17.8)	-

*SD* Standard deviation, *WPAI-OA* Work Productivity and Activity Impairment-Osteoarthritis

^a^For participants aged 65 years and younger

**Fig. 2:**
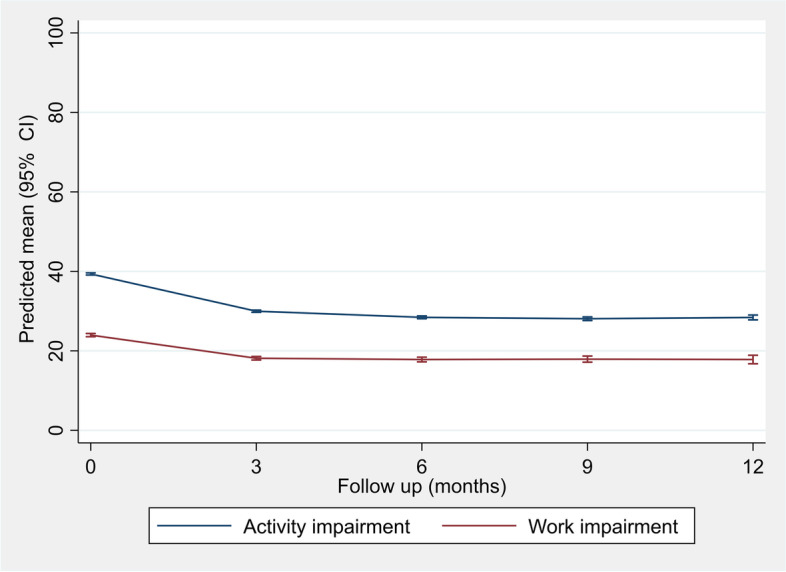
Predicted mean (95% confidence interval) in work and activity impairments at the baseline and follow ups. Estimates obtained from random intercept model adjusted for sociodemographic and health-related characteristics as well as the baseline value of the outcome of interest

After adjustment for the baseline characteristics, work impairment declined by 5.8 (95% CI: 5.3, 6.4), 6.1 (5.5, 6.8), 6.0 (5.2, 6.9) and 6.1 (5.0, 7.3) percentage points at the 3, 6, 9, and 12 months follow ups compared with baseline (Fig. 2, Table A1 in Additional file 1). The corresponding reductions in activity impairment were 9.4 (95% CI: 9.0, 9.7), 10.9 (10.5, 11.3), 11.3 (10.8, 11.8), and 10.9 (10.3, 11.6), respectively. Excluding those with adherence < 80%, and limiting the sample to those with complete follow up responses had almost no effect on these estimates (Table A1 in Additional file 1). Subgroup analyses showed that females experienced greater improvements than males in both activity and work impairments, even though these improvements were statistically inconclusive (Table A2 in Additional file 1). Participants with knee OA had greater improvements than those with hip OA. The participants aged 75 + years experienced 2.6 (95% CI: 1.1, 4.2), 3.8 (1.9, 5.8), 3.5 (1.0, 5.9), and 4.8 (1.5, 8.0) lower percentage point improvements at activity impairments at 3, 6, 9, and 12 months follow ups, compared with those aged 24–50 years (Table A3 in Additional file 1). No conclusive differences were seen in changes in work impairments across age groups.

**Fig. 3:**
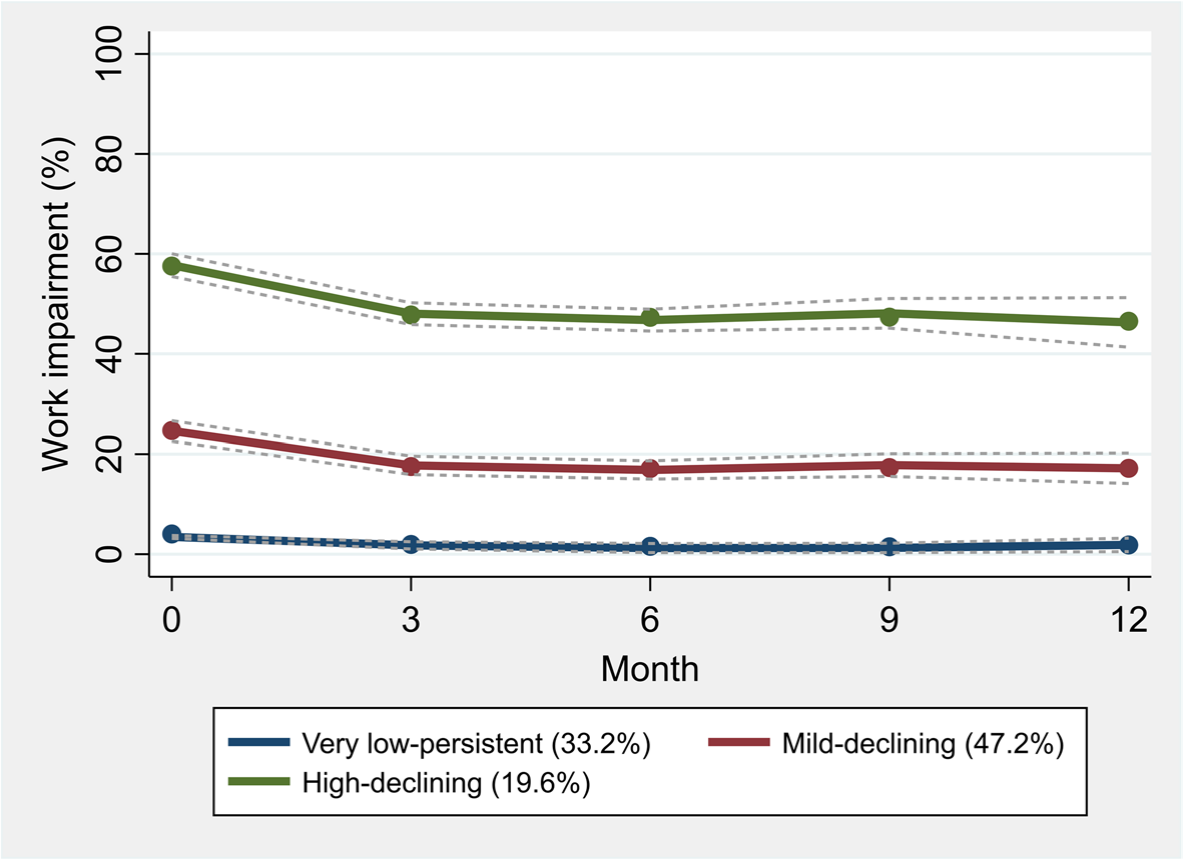
Trajectory classes of work impairment from baseline to 1-year follow up

GBTM identified 3 classes with distinct trajectories in work impairment (Fig. 3, see Table A4 in Additional file 1 for more details on model and fit statistics and Fig. A1 for individual trajectories within each class): 33.2% with very low work impairment at baseline which was persistent over follow up (“very low–persistent”), 47.2% with mild impairment at baseline which were declining during follow up (“mild–declining”), and 19.6% with high impairment at baseline with declining trajectory over follow up (“high–declining”). Compared to other classes, participants in “high–declining” class had, on average, less education, higher BMI, more comorbidities, and worse patient-reported outcomes at baseline (Table A5 in Additional file 1). The results of multinomial logistic regression showed that male sex, higher BMI, lower education, walking difficulties, rheumatoid arthritis, and worse patient-reported outcomes were generally associated with higher probability of being assigned to “high–declining” class than other classes (Table 2). Dominance analysis suggested that pain followed by education and BMI were most important predictors of trajectory class membership. However, PDI, MCC and CEN suggested poor predictive ability of the baseline characteristics to accurately classify the participants into trajectory classes with only 56.9% of them being accurately assigned to their trajectory class (Fig. A2 in Additional file 1).

**Table 2:** Relative risk ratios (95% confidence intervals) for work impairment trajectory class membership from multinomial logit model and the relative importance in predicting trajectory class membership from dominance analysis

Variable	VL–P vs. H–D	M–D vs. H–D	Dominance statistics^a^	Ranking^b^
Female sex (male = ref)	1.11 (0.89, 1.38)	1.23 (1.01, 1.49)	0.0006	10
Age (24–50 years = ref)	1.0	1.0	0.0011	9
51–60 years	0.94 (0.73, 1.23)	1.13 (0.90, 1.42)		
61–65 years	1.05 (0.78, 1.41)	1.32 (1.01, 1.71)		
Hip as index joint (knee = ref)	0.82 (0.67, 0.99)	1.23 (1.04, 1.45)	0.0033	7
Education (Less than high school = ref)	1.0	1.0	0.0144	2
High school	2.24 (1.37, 3.66)	1.23 (0.88, 1.74)		
College/university	4.96 (3.04, 8.11)	1.85 (1.31, 2.61)		
Body mass index (< 25 = ref)	1.0	1.0	0.0127	3
25–29	0.68 (0.54, 0.86)	0.92 (0.74, 1.14)		
30–34	0.48 (0.37, 0.64)	0.67 (0.52, 0.85)		
35 +	0.39 (0.27, 0.59)	0.79 (0.58, 1.08)		
Diabetes	1.19 (0.70, 2.01)	0.88 (0.57, 1.36)	0.0003	12
Lung diseases	1.18 (0.83, 1.68)	0.95 (0.70, 1.30)	0.0004	11
Balance troubles	1.22 (0.59, 2.49)	1.14 (0.64, 2.01)	0.0002	14
Rheumatoid arthritis	0.50 (0.28, 0.90)	0.70 (0.45, 1.09)	0.0015	8
Cardiovascular diseases	0.91 (0.51, 1.64)	1.12 (0.68, 1.85)	0.0002	13
Walking difficulties	0.44 (0.29, 0.68)	0.81 (0.58, 1.13)	0.0046	6
General health	1.24 (1.18, 1.31)	1.10 (1.05, 1.15)	0.0122	4
Pain	0.47 (0.44, 0.50)	0.61 (0.58, 0.64)	0.0869	1
Physical function	1.04 (1.02, 1.06)	1.01 (0.99, 1.03)	0.0062	5
McFadden pseudo-*R*^2^	0.145			
MCC	0.271			
CEN	0.581			
PDI	0.600			

*CEN* Confusion entropy, *H–D* High–declining, *VL–P* Very low–persistent, *MCC* Matthews correlation coefficient, *M–D* Mild–declining, *PDI* Polytomous discrimination index

^a^Each variable absolute contribution to McFadden pseudo-*R*^2^

^b^The relative importance of each variable in predicting trajectory class membership

**Fig. 4:**
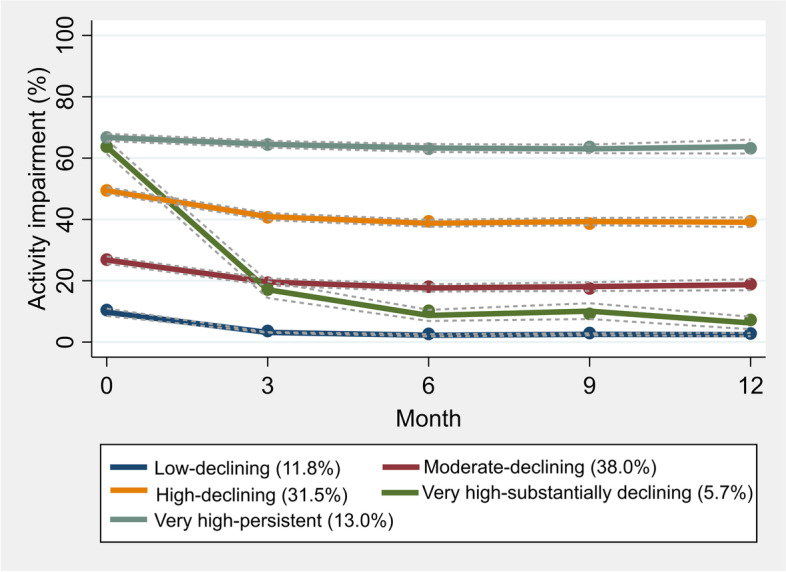
Trajectory classes of activity impairment from baseline to 1-year follow up

For activity impairment, we identified 5 classes with distinct trajectories (Fig. 4, for more details see Table A4 and Fig. A3 in Additional file 1): 11.8% with low baseline impairment and a declining trajectory (“low–declining”), 38.0% with moderate baseline impairment and declining trajectory (“moderate–declining”), 31.5% with high baseline impairment and a declining trajectory (“high–declining”), 5.7% with very high impairment at baseline and substantial reductions in impairment over follow up (“very high–substantially declining”), and 13.0% with very high and persistent impairment during follow up (“very high–persistent”). A larger proportion of individuals in the class “very high–substantially declining” reported knee as their index joint compared with other classes (Table A6 in Additional file 1). On the other hand, compared to other classes, individuals in the class “very high–persistent” had generally lower education, higher BMI, weren’t working, had coexisting conditions, and reported poorer patient-reported outcomes at baseline. The results of multinomial logistic regression showed that male sex, higher BMI, poorer baseline patient reported outcomes, walking difficulties, and not working/being retired were generally associated with higher probability of being assigned to the class “very high–persistent” instead of other classes (Table 3). Dominance analysis suggested that pain at baseline was the most important predictor of trajectory class membership. All predictive accuracy measures suggested poor predictive ability of baseline characteristics to accurately predict the trajectory class membership. Indeed, only about half (51.3%) of the subjects were accurately assigned to their trajectory class and no one was assigned to the smallest class (i.e. “very high–substantially declining”, Fig. A4 in Additional file 1).

**Table 3:** Relative risk ratios (95% confidence intervals) for activity impairment trajectory class membership from multinomial logit model and the relative importance in predicting trajectory class membership from dominance analysis

Variable	L–D vs. VH–P	M–D vs. VH–P	H–D vs. VH–P	VH–SD vs. VH–P	Dominance statistics^a^	Ranking^b^
Female sex (male = ref)	1.65 (1.37, 1.99)	1.39 (1.20, 1.61)	1.15 (1.00, 1.32)	1.20 (0.96, 1.51)	0.0004	12
Age (24–50 years = ref)	1.00	1.00	1.00	1.00	0.0008	9
51–65 years	0.90 (0.67, 1.23)	0.99 (0.77, 1.27)	0.97 (0.77, 1.22)	0.87 (0.60, 1.24)		
66–74 years	0.99 (0.67, 1.45)	1.08 (0.79, 1.47)	0.94 (0.71, 1.26)	0.76 (0.48, 1.20)		
75 + years	0.69 (0.44, 1.07)	0.90 (0.63, 1.27)	0.94 (0.68, 1.30)	0.51 (0.30, 0.87)		
Hip as index joint (knee = ref)	1.03 (0.88, 1.20)	0.90 (0.80, 1.02)	0.83 (0.74, 0.93)	0.50 (0.41, 0.61)	0.0015	8
Education (Less than high school = ref)	1.00	1.00	1.00	1.00	0.0016	7
High school	1.23 (0.89, 1.71)	0.99 (0.79, 1.23)	0.97 (0.79, 1.19)	1.11 (0.77, 1.60)		
College/university	1.21 (0.88, 1.66)	0.98 (0.79, 1.22)	0.98 (0.80, 1.20)	1.38 (0.97, 1.98)		
Employment (Working = ref)	1.00	1.00	1.00	1.00	0.0033	6
Not working	0.32 (0.21, 0.48)	0.42 (0.32, 0.55)	0.67 (0.53, 0.84)	0.65 (0.43, 0.97)		
Retired	0.44 (0.34, 0.57)	0.59 (0.49, 0.73)	0.85 (0.71, 1.02)	0.94 (0.70, 1.26)		
Body mass index (< 25 = ref)	1.00	1.00	1.00	1.00	0.0083	3
25–29	0.72 (0.60, 0.87)	0.83 (0.71, 0.97)	0.96 (0.83, 1.12)	0.72 (0.58, 0.89)		
30–34	0.56 (0.44, 0.72)	0.64 (0.53, 0.77)	0.87 (0.73, 1.04)	0.44 (0.33, 0.60)		
35 +	0.24 (0.16, 0.36)	0.36 (0.28, 0.46)	0.64 (0.52, 0.79)	0.37 (0.25, 0.55)		
Diabetes	1.53 (1.06, 2.22)	1.30 (1.00, 1.68)	1.04 (0.82, 1.31)	0.96 (0.61, 1.50)	0.0003	14
Lung diseases	1.61 (1.22, 2.13)	1.42 (1.15, 1.76)	1.23 (1.01, 1.50)	1.37 (0.99, 1.90)	0.0003	13
Balance troubles	1.01 (0.60, 1.69)	1.13 (0.81, 1.56)	1.05 (0.79, 1.39)	0.79 (0.44, 1.43)	0.0004	11
Rheumatoid arthritis	0.80 (0.53, 1.21)	0.86 (0.65, 1.14)	0.91 (0.71, 1.18)	0.73 (0.45, 1.19)	0.0005	10
Cardiovascular diseases	1.12 (0.80, 1.55)	1.10 (0.87, 1.39)	1.01 (0.82, 1.25)	1.10 (0.76, 1.59)	0.0003	15
Walking difficulties	0.24 (0.17, 0.35)	0.43 (0.35, 0.54)	0.65 (0.54, 0.79)	0.56 (0.39, 0.80)	0.0054	4
General health	1.62 (1.54, 1.70)	1.29 (1.25, 1.34)	1.10 (1.06, 1.13)	1.24 (1.18, 1.31)	0.0177	2
Pain	0.28 (0.26, 0.29)	0.40 (0.38, 0.42)	0.65 (0.62, 0.68)	0.72 (0.68, 0.77)	0.1127	1
Physical function	1.06 (1.04, 1.08)	1.05 (1.03, 1.06)	1.03 (1.01, 1.04)	1.03 (1.00, 1.05)	0.0050	5
McFadden pseudo-*R*^2^	0.159					
MCC	0.264					
CEN	0.462					
PDI	0.435					

*CEN* Confusion entropy, *H–D* High–declining, *L–D* Low–declining, *MCC* Matthews correlation coefficient, *M–D* Moderate–declining, *PDI* Polytomous discrimination index, *VH–P* Very high–persistent, *VH–SD* Very high–substantially declining

^a^Each variable absolute contribution to McFadden pseudo-*R*^2^

^b^The relative importance of each variable in predicting trajectory class membership

## Discussion

Participation in a digital self-management program was associated with improvements in work and activity impairments among individuals with hip or knee OA, with the greatest improvements reported at 3-month follow up. These observed improvements were not sensitive to adherence level or loss to follow ups. However, there were important variations in trajectory of these outcomes with 3 and 5 distinct trajectory classes of work and activity impairments, respectively. While pain at baseline was the most important predictor of trajectory class membership, the overall predictive accuracy of the baseline characteristics was poor.

Our results confirmed previous findings on the higher contribution of presenteeism (reduced productivity while working) than absenteeism (work absence) to the overall lost productivity costs attributable to OA [28–30], highlighting the importance of capturing presenteeism in OA cost-of-illness studies. The levels of work [24.3%] and activity [39.3%] impairments reported at baseline in our study were similar to the levels reported for mild to moderate OA [31, 32]. While the magnitude of work impairment was lower [24.3% vs. 28.6%] than the one reported in a sample of OA subjects across five European countries (i.e. France, Germany, UK, Italy, and Spain), the level of activity impairment was almost identical [39.3% vs. 39.8%] [28].

Although a minimal clinically important difference (MCID) for WPAI of 7% for Crohn’s disease [33] and 20% for psoriasis [34] and psoriatic arthritis [35] was reported, there is no published MCID for WPAI-OA, hindering the interpretation of the observed improvements in our study in terms of clinical importance. To our knowledge, only one previous study investigated the effect of self-management on work and activity impairment using WPAI–OA and reported negligible changes in these outcomes following face-to-face self-management with/without self-monitoring physical activity in persons with OA [29]. The cross-study differences in sociodemographic, clinical characteristics and the levels of work/activity impairments might explain the discrepancy with our findings. For instance, there were milder presenteeism [19.3% vs. 24.3%] and activity impairment [30.0% vs. 39.3%] in Östlind’s study [29] compared to our study. These milder impairments might have limited the possibility for improvement, especially considering that the cross-study differences [i.e. 5% for presenteeism and 9% for activity impairment] are close to the improvements observed in the present study. On the other hand, a few studies used sick leave and disability pension as measures of work impairment and, consistent with our findings, reported improvements in these outcomes following participation in face-to-face [36, 37] or digital self-management for OA [38]. For instance, participating in face-to-face self-management programs for OA in Sweden and Denmark was associated with reductions in proportion of people on sick leave from 13.6% and 24.3% at baseline to 5.1% and 14.9% at 1-year follow up, respectively [36, 37].

The group-based trajectory modelling revealed substantial variations in trajectories of work and activity impairments. Indeed, while the mean baseline activity/work impairments in our sample were similar to the ones reported for people with mild to moderate OA, 20%/50% of participants in our sample had work/activity impairments similar to that reported in persons with severe OA [31, 32]. Among 2,421 participants with very high level of activity impairments [≥ 60%] at baseline, 27% experienced substantial improvements in their impairments and remaining individuals experienced very little improvement. Participants in trajectory subgroups with high levels of impairments had generally higher BMI, more co-existing conditions, and worse patient-reported outcomes at baseline. While, to our knowledge, no previous study explored heterogeneity in work/activity impairments measured by WPAI among people with OA, a study among persons with an incident sick leave spell due to OA also identified important heterogeneity in trajectory of sick leave/disability pension days in Sweden [39]. Compared to other subgroups, the subgroup with “late decrease” in sick leave/disability pension days tended to have lower education, higher unemployment, and more serious morbidity [39]. These characteristics are similar to those of the “very high–persistent” subgroup in activity impairment and “high–declining” subgroup in work impairment in the present study.

Our dominance analysis suggested that pain at baseline was the most important predictor of work/activity impairments. This is consistent with previous studies that identified pain as an important predictor of productivity loss and disability pension among persons with OA [40, 41]. About half of the effect of pain on the onset of work productivity loss in OA may be explained by physical limitation [40]. While these findings highlight the importance of pain relief in OA management, the poor predictive ability of pain and other sociodemographic and clinical characteristics in predicting trajectories of work/activity impairment in the present study discourage any intervention targeting only those with high pain at baseline. Consistent with our findings, a study in Sweden also reported poor to moderate predictive ability of sociodemographic and patient history information obtained from registers in predicting the duration of sick leave due to knee OA [42]. Given the importance of person-centred care, our findings highlight the need for further research to more accurately predict the participants’ work/activity impairments trajectories.

We acknowledge several limitations of the current study. The data used in the study are self-reported and prone to biases. While 7-day recall period has been suggested as an adequate period for recall accuracy of work impairment [19], the possibility of recall bias in responses to WPAI cannot be fully ruled out. The digital program is integrated in the Swedish healthcare system and hence accessible to all people seeking care for OA, but the possibility of a self-selected sample cannot be excluded. For instance, among persons with a knee or hip OA diagnosis between 1998 and 2016 in Southern Sweden, around 59% were females and 21% had a college/university level education [43]. The corresponding proportions in the present study were 76% and 56%, respectively. This suggests that our findings might not be generalizable to the general OA population. The lack of a control group means that the observed improvements cannot be fully attributed to the intervention and possibility for other factors such as natural course of disease or context effects cannot be ruled out. We didn’t have data on some potentially important confounders including type of occupation, health behaviours, cognitive function, and medication use. The identified trajectory subgroups should be treated as an approximation of a more complex underlying reality, not as “real entities” present within the population [21].

## Conclusion

The present study showed improvements in work/activity impairments being associated with participation in a digital self-management program for OA. We also observed important variations in trajectories of work/activity impairments with pain at baseline being the most important predictor of these trajectories. However, poor predictive ability of the baseline characteristics in identifying trajectory classes calls for further research and collecting more detailed data.

## Supplementary Information

**Additional file 1: Table A1.** Predicted mean change (95% confidence interval) in work and activity impairments compared to the baseline among participants. **Table A2.** Predicted mean change and difference in mean change (95% confidence interval) in work and activity impairments by sex and index joint. **Table A3.** Predicted mean change and difference in mean change (95% confidence interval) in work and activity impairments by age group. **Table A4.** Parameters estimated and model fit measures for latent class trajectories of work and activity impairments. **Table A5.** Baseline characteristics of work impairment trajectory classes. **Table A6.** Baseline characteristics of activity impairment trajectory classes. **Figure A1.** Individual trajectories of participants within work impairment trajectory classes. **Figure A2.** Confusion matrix of the actual work impairment trajectory class and the predicted class using the baseline characteristics. **Figure A3.** Individual trajectories of participants within activity impairment trajectory classes. **Figure A4.** Confusion matrix of the actual activity impairment trajectory class and the predicted class using the baseline characteristics.

## Data Availability

The data that support the findings of this study are available from Joint Academy® but restrictions apply to the availability of these data, which were used under ethical permission for the current study, and so are not publicly available. Data may be made available through the corresponding author upon reasonable request and with permission of Joint Academy®.

## Abbreviations

MCID: Minimal clinically important difference
OA: Osteoarthritis
PDI: Polytomous discrimination index
MCC: Matthews correlation coefficient
CEN: Confusion entropy
GBTM: Group-based trajectory modelling
BOA: Better management of patients with OsteoArthritis
WPAI–OA: Work Productivity and Activity Impairment–Osteoarthritis
