# Supplementary material for: Digital self-management of hip and knee osteoarthritis and trajectories of work and activity impairments
Source: BMC Musculoskelet Disord. 2023 Mar 18;24:207. doi: 10.1186/s12891-023-06322-z (PMC10024026; doi:10.1186/s12891-023-06322-z)

Table A1. Predicted mean change (95% confidence interval) in work and activity impairments compared to the baseline among participants. Estimates obtained from random intercept model adjusted for sociodemographic and health-related characteristics as well as the baseline value of the outcome of interest.

	All	Complete follow up responses	Adherence ≥80% at 3-month follow up
Work impairment %			
3-month	-5.8 (-6.4, -5.3)	-6.2 (-7.7, -4.7)	-6.2 (-6.9, -5.5)
6-month	-6.1 (-6.8, -5.5)	-6.3 (-7.8, -4.9)	-6.5 (-7.3, -5.7)
9-month	-6.0 (-6.9, -5.2)	-7.0 (-8.5, -5.5)	-6.4 (-7.4, -5.4)
12-month	-6.1 (-7.3, -5.0)	-6.3 (-7.8, -4.8)	-6.6 (-7.8, -5.3)
Sample size	5186	872	3607
Activity impairment %			
3-month	-9.4 (-9.7, -9.0)	-10.0 (-10.8, -9.2)	-9.9 (-10.3, -9.5)
6-month	-10.9 (-11.3, -10.5)	-11.4 (-12.2, -10.6)	-11.6 (-12.0, -11.1)
9-month	-11.3 (-11.8, -10.8)	-12.1 (-12.9, -11.3)	-11.7 (-12.3, -11.2)
12-month	-10.9 (-11.6, -10.3)	-11.2 (-12.1, -10.4)	-11.6 (-12.3, -10.9)
Sample size	14676	2609	10977

Table A2. Predicted mean change and difference in mean change (95% confidence interval) in work and activity impairments by sex and index joint. Estimates obtained from random intercept model adjusted for sociodemographic and health-related characteristics as well as the baseline value of the outcome of interest.

	Sex			Index joint		
	Males	Females	Difference [a]	Knee	Hip	Difference [b]
Work impairment %						
3-month	-4.7 (-5.9, -3.6)	-6.2 (-6.8, -5.5)	-1.5 (-2.8, -0.1)	-6.5 (-7.2, -5.8)	-4.8 (-5.7, -3.9)	1.6 (0.5, 2.8)
6-month	-5.0 (-6.3, -3.6)	-6.5 (-7.3, -5.7)	-1.6 (-3.1, 0.0)	-7.2 (-8.1, -6.3)	-4.5 (-5.6, -3.4)	2.8 (1.4, 4.2)
9-month	-4.8 (-6.5, -3.1)	-6.5 (-7.4, -5.5)	-1.7 (-3.6, 0.3)	-6.6 (-7.7, -5.5)	-5.2 (-6.5, -3.8)	1.4 (-0.3, 3.2)
12-month	-4.4 (-6.7, -2.1)	-6.7 (-8.0, -5.4)	-2.3 (-4.9, 0.4)	-7.1 (-8.5, -5.6)	-4.7 (-6.5, -2.9)	2.4 (0.1, 4.7)
Activity impairment %						
3-month	-9.0 (-9.7, -8.3)	-9.5 (-9.9, -9.1)	-0.5 (-1.3, 0.3)	-10.5 (-10.9, -10.0)	-7.7 (-8.3, -7.2)	2.7 (2.0, 3.4)
6-month	-10.6 (-11.5, -9.8)	-11.0 (-11.5, -10.5)	-0.4 (-1.3, 0.6)	-12.2 (-12.7, -11.7)	-9.0 (-9.6, -8.3)	3.3 (2.4, 4.1)
9-month	-10.2 (-11.2, -9.2)	-11.6 (-12.2, -11.1)	-1.4 (-2.6, -0.3)	-12.6 (-13.2, -12.0)	-9.2 (-10.0, -8.4)	3.4 (2.4, 4.4)
12-month	-10.1 (-11.4, -8.8)	-11.2 (-11.9, -10.5)	-1.1 (-2.6, 0.4)	-12.2 (-13.0, -11.4)	-9.0 (-10.0, -7.9)	3.3 (1.9, 4.6)

[a] Females vs. males.

[b] Hip osteoarthritis vs. knee osteoarthritis.

Table A3. Predicted mean change and difference in mean change (95% confidence interval) in work and activity impairments by age group. Estimates obtained from random intercept model adjusted for sociodemographic and health-related characteristics as well as the baseline value of the outcome of interest.

	Age groups				Differences in mean change (the 24-50 years age group as reference)		
	24-50 years	51-65 years	66-74 years [a]	75+ years [a]	Difference1 [b]	Difference2 [c]	Difference3 [d]
Work impairment %							
3-month	-5.8 (-7.2, -4.4)	-5.8 (-6.5, -5.2)	NA	NA	-0.1 (-1.6, 1.4)	NA	NA
6-month	-4.8 (-6.5, -3.1)	-6.4 (-7.1, -5.6)	NA	NA	-1.6 (-3.4, 0.3)	NA	NA
9-month	-7.0 (-9.2, -4.7)	-5.9 (-6.8, -5.0)	NA	NA	1.1 (-1.3, 3.5)	NA	NA
12-month	-6.6 (-9.8, -3.5)	-6.1 (-7.3, -4.9)	NA	NA	0.6 (-2.8, 3.9)	NA	NA
Activity impairment %							
3-month	-10.8 (-12.0, -9.5)	-9.5 (-10.0, -9.0)	-9.4 (-10.0, -8.8)	-8.1 (-9.1, -7.1)	1.3 (-0.1, 2.6)	1.4 (-0.0, 2.8)	2.6 (1.1, 4.2)
6-month	-12.9 (-14.5, -11.4)	-11.3 (-11.9, -10.7)	-10.6 (-11.3, -9.9)	-9.1 (-10.3, -7.9)	1.6 (-0.0, 3.3)	2.3 (0.6, 4.0)	3.8 (1.9, 5.8)
9-month	-12.5 (-14.4, -10.6)	-11.8 (-12.5, -11.1)	-11.1 (-11.9, -10.2)	-9.0 (-10.5, -7.5)	0.7 (-1.4, 2.8)	1.4 (-0.7, 3.6)	3.5 (1.0, 5.9)
12-month	-12.3 (-15.0, -9.7)	-12.3 (-13.2, -11.3)	-10.0 (-11.1, -9.0)	-7.6 (-9.5, -5.7)	0.1 (-2.7, 2.9)	2.3 (-0.5, 5.2)	4.8 (1.5, 8.0)

[a] Work impairment was analysed only among people aged 65 years and younger.

[b] 51-65 years vs. 24-50 years.

[c] 66-74 years vs. 24-50 years.

[d] 75+ years vs. 24-50 years.

Table A4. Parameters estimated and model fit measures for latent class trajectories of work and activity impairments.

	Activity impairment					Work impairment		
	Low- declining	Moderate-declining	High-declining	Very high-substantially declining	Very high-persistent	Very low-persistent	Moderate-declining	High-declining
**Model coefficients (se)**								
Intercept	5.82 (0.68)	26.42 (0.46)	49. 40 (0.053)	63.77 (1.04)	66.87 (0.52)	-12.56 (1.15)	23.23 (0.78)	57.89 (0.95)
Linear term	-7.10 (0.65)	-3.80 (0.27)	-4.28 (0.31)	-24.65 (0.96)	-0.92 (0.21)	-3.30 (0.48)	-4.48 (0.51)	-5.39 (0.77)
Quadratic term	0.93 (0.015)	0.45 (0.06)	0.55 (0.07)	3.29 (0.20)	0.06 (0.02)	0.22 (0.04)	0.65 (0.12)	0.81 (0.18)
Cubic term	-0.04 (0.01)	-0.02 (0.00)	-0.02 (0.00)	-0.14 (0.01)			-0.03 (0.01)	-0.04 (0.01)
**Model fit measures**								
Group membership probability	11.8%	38.0%	31.5%	5.7%	13.0%	33.2%	47.2%	19.6%
Average posterior probability	0.811	0.805	0.746	0.707	0.826	0.864	0.834	0.876
Odds of correct classification	32.0	6.7	6.4	40.2	31.9	12.8	5.6	29.0
Entropy statistics	0.677					0.679		
Bayesian information criterion (BIC)	-183156.2					-45389.5		

Figure A1. Individual trajectories of participants within work impairment trajectory classes.

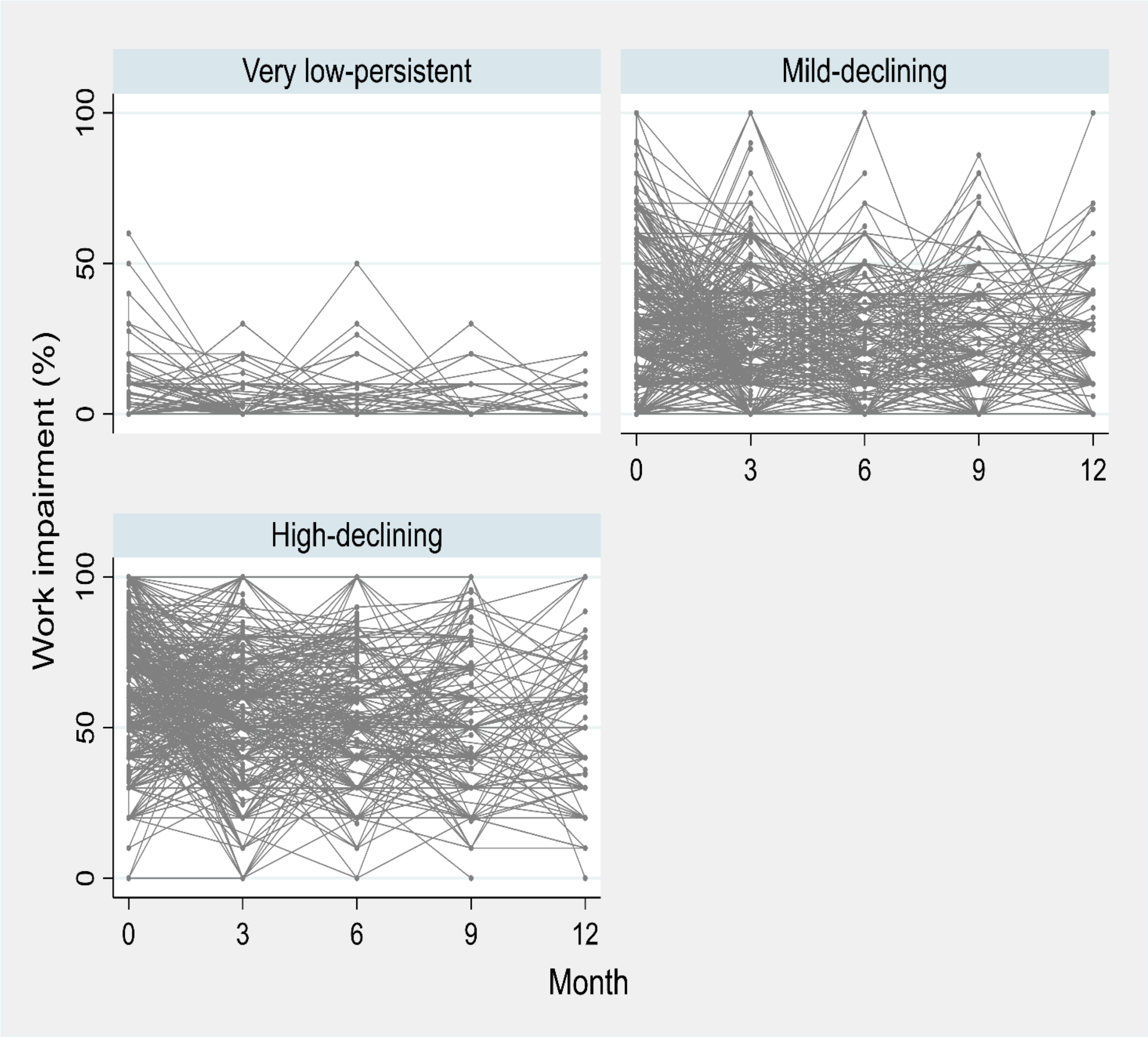

Table A5. Baseline characteristics of work impairment trajectory classes

	Very low-persistent (n=1,748)	Moderate-declining (n=2,512)	High-declining (n=926)
Female, n (%)	1,305 (74.7)	1,957 (77.9)	701 (75.7)
Age, mean (SD)	56.2 (6.2)	56.6 (5.9)	56.0 (5.8)
Index joint, n (%)			
Knee	1,138 (65.1)	1,427 (56.8)	581 (62.7)
Hip	610 (34.9)	1,085 (43.2)	345 (37.3)
Education, n (%)			
Less than high school	31 (1.7)	116 (4.6)	70 (7.6)
High school	541 (31.0)	1,058 (42.1)	500 (54.0)
College/university	1,176 (67.3)	1,338 (53.3)	356 (38.4)
Body mass index, mean (SD)	26.3 (4.3)	27.8 (5.1)	29.6 (5.5)
Diabetes, n (%)	45 (2.6)	79 (3.1)	46 (5.0)
Lung diseases, n (%)	143 (8.2)	233 (9.3)	110 (11.9)
Balance troubles, n (%)	21 (1.2)	48 (1.9)	24 (2.6)
Rheumatoid arthritis, n (%)	28 (1.6)	80 (3.2)	52 (5.6)
Cardiovascular diseases, n (%)	40 (2.3)	81 (3.2)	26 (2.8)
Walking difficulties, n (%)	71 (4.1)	236 (9.4)	145 (15.7)
General health, mean (SD)	7.0 (1.7)	6.4 (1.8)	5.9 (1.9)
Pain, mean (SD)	4.2 (1.9)	5.2 (1.7)	6.6 (1.5)
Physical function, mean (SD)	14.2 (4.5)	13.1 (4.4)	12.2 (4.1)
Work impairment in %, mean (SD)	2.8 (6.4)	25.6 (17.0)	61.2 (19.5)
Activity impairment in %, mean (SD)	25.1 (22.7)	38.4 (21.0)	59.4 (18.2)

Figure A2. Confusion matrix of the actual work impairment trajectory class and the predicted class using the baseline characteristics.

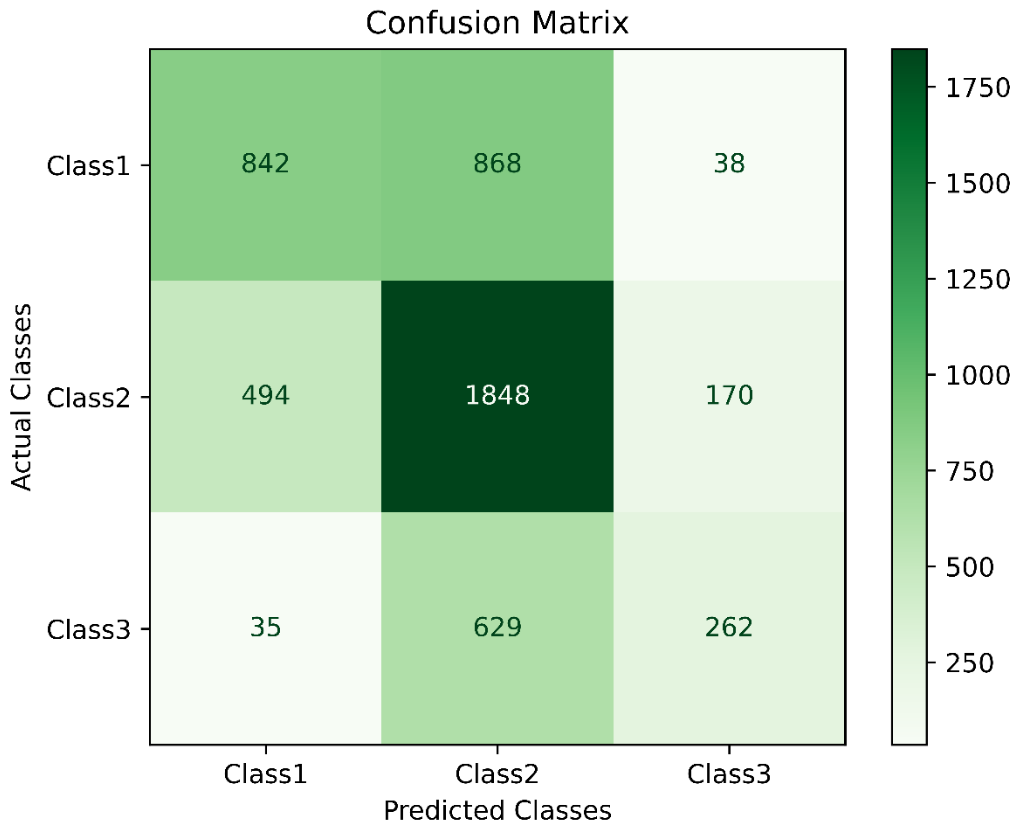

Figure A3. Individual trajectories of participants within activity impairment trajectory classes.

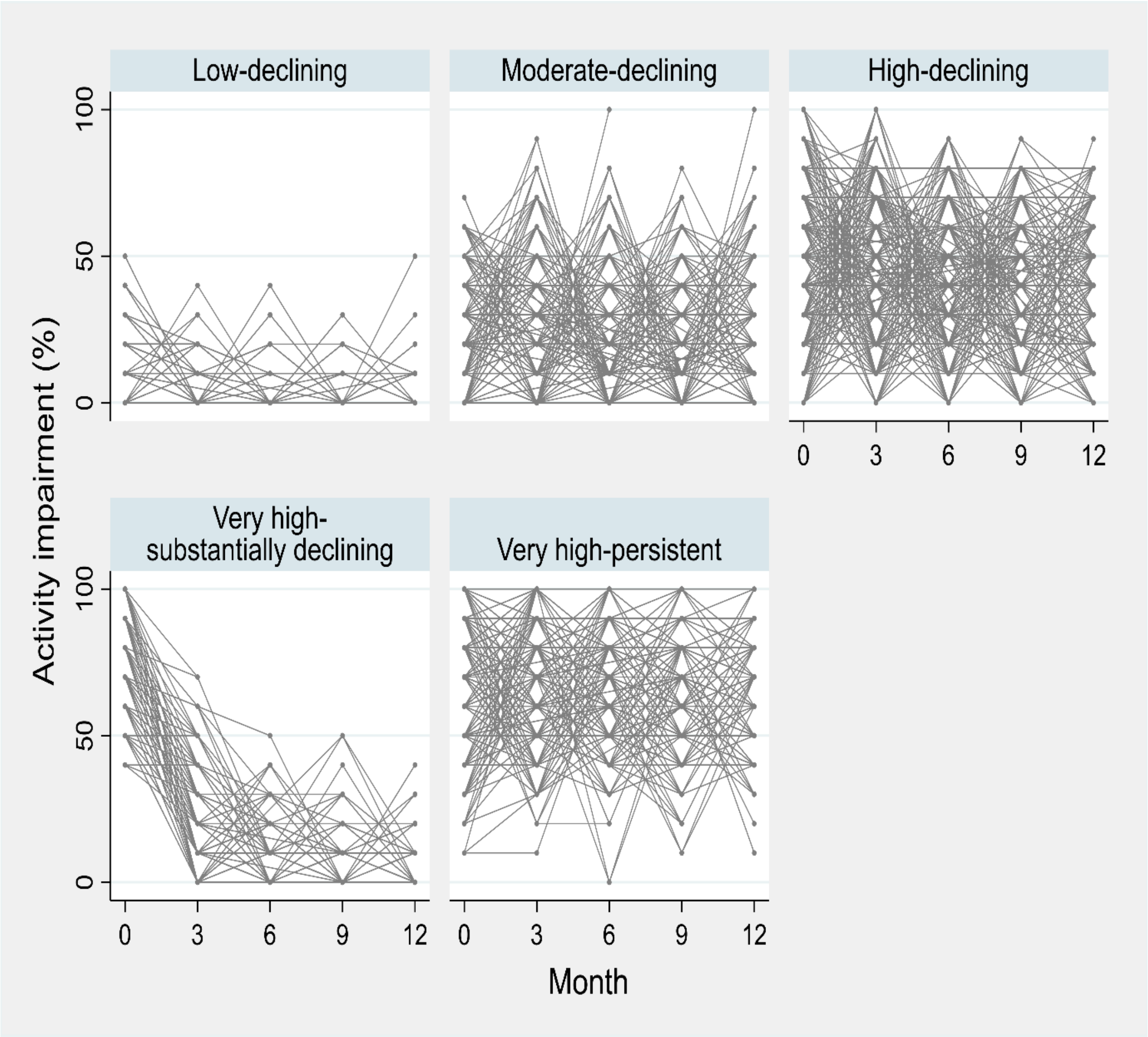

Table A6. Baseline characteristics of activity impairment trajectory classes

	Low-declining (n=1,705)	Moderate-declining (n=5,649)	High-declining (n=4,901)	Very high-substantially declining (n=651)	Very high-persistent (n=1,770)
Female, n (%)	1,300 (76.3)	4,255 (75.3)	3,686 (75.2)	498 (76.5)	1,340 (75.7)
Age, mean (SD)	63.3 (9.2)	64.3 (9.0)	64.3 (9.1)	63.4 (8.9)	63.7 (9.4)
Index joint, n (%)					
Knee	967 (56.7)	3,354 (59.4)	2,997 (61.1)	467 (71.7)	1,016 (57.4)
Hip	738 (43.3)	2,295 (40.6)	1,904 (38.9)	184 (28.3)	754 (42.6)
Education, n (%)					
Less than high school	81 (4.8)	413 (7.3)	458 (9.4)	45 (6.9)	187 (10.6)
High school	529 (31.0)	1,909 (33.8)	1,865 (38.0)	219 (33.6)	747 (42.2)
College/university	1,095 (64.2)	3,327 (58.9)	2,578 (52.6)	387 (59.5)	836 (47.2)
Body mass index, mean (SD)	25.6 (4.0)	26.5 (4.2)	27.9 (4.9)	26.7 (4.5)	29.1 (5.6)
Employment, n (%)					
Working	898 (52.7)	2,599 (46.0)	2,032 (41.5)	279 (42.9)	673 (38.0)
Not working	44 (2.6)	196 (3.5)	274 (5.6)	33 (5.1)	170 (9.6)
Retired	763 (44.7)	2,854 (50.5)	2,595 (52.9)	339 (52.0)	927 (52.4)
Diabetes, n (%)	65 (3.8)	282 (5.0)	297 (6.1)	28 (4.3)	150 (8.5)
Lung diseases, n (%)	146 (8.6)	561 (9.9)	561 (11.5)	70 (10.8)	221 (12.5)
Balance troubles, n (%)	27 (1.6)	158 (2.8)	189 (3.9)	15 (2.3)	100 (5.7)
Rheumatoid arthritis, n (%)	46 (2.7)	221 (3.9)	253 (5.2)	23 (3.5)	126 (7.1)
Cardiovascular diseases, n (%)	85 (5.0)	384 (6.8)	395 (8.1)	45 (6.9)	175 (9.9)
Walking difficulties, n (%)	62 (3.6)	493 (8.7)	751 (15.3)	72 (11.1)	428 (24.2)
General health, mean (SD)	7.5 (1.7)	6.8 (1.7)	6.2 (1.8)	6.8 (1.9)	5.8 (2.0)
Pain, mean (SD)	3.3 (1.8)	4.4 (1.7)	5.8 (1.6)	6.0 (1.8)	6.8 (1.5)
Physical function, mean (SD)	14.0 (4.3)	13.2 (4.2)	12.3 (4.3)	12.5 (4.8)	11.4 (4.3)
Activity impairment in %, mean (SD)	8.9 (10.3)	25.8 (13.2)	50.8 (15.7)	69.5 (12.0)	68.9 (14.3)

Figure A4. Confusion matrix of the actual activity impairment trajectory class and the predicted class using the baseline characteristics.

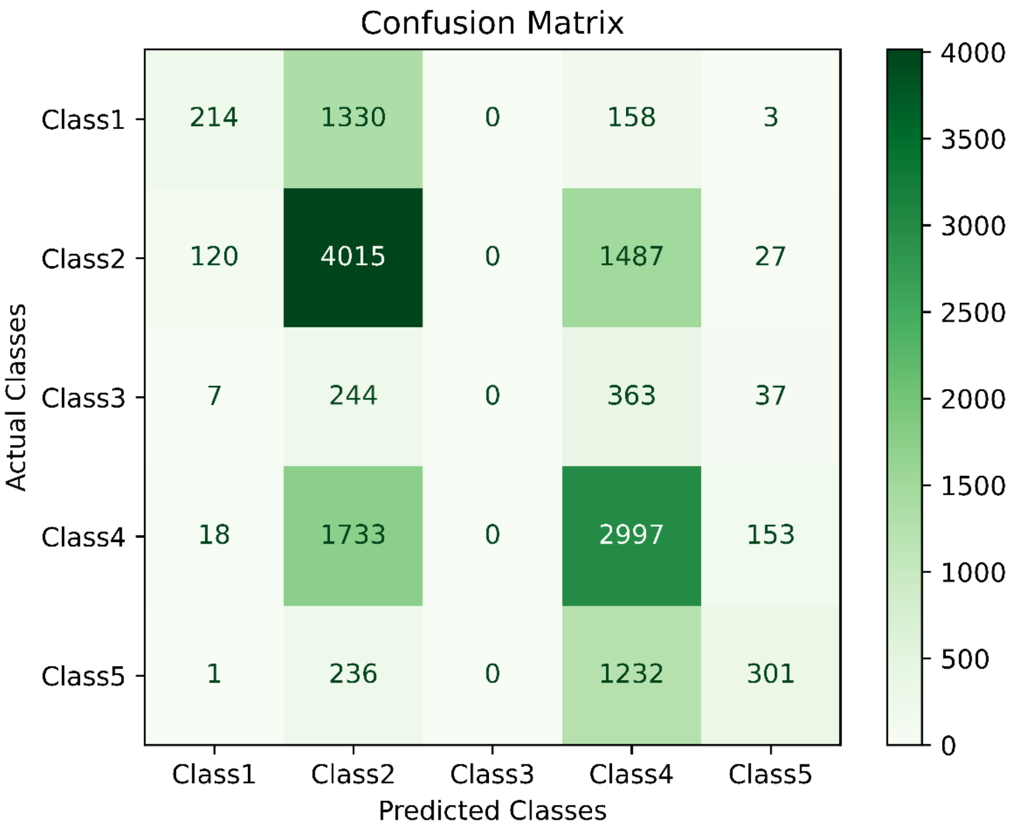

Supplement: Supplementary file 1 — Additional file 1: Table A1. Predicted mean change (95% confidence interval) in work and activity impairments compared to the baseline among participants. Table A2. Predicted mean change and difference in mean change (95% confidence interval) in work and activity impairments by sex and index joint. Table A3. Predicted mean change and difference in mean change (95% confidence interval) in work and activity impairments by age group. Table A4. Parameters estimated and model fit measures for latent class trajectories of work and activity impairments. Table A5. Baseline characteristics of work impairment trajectory classes. Table A6. Baseline characteristics of activity impairment trajectory classes. Figure A1. Individual trajectories of participants within work impairment trajectory classes. Figure A2. Confusion matrix of the actual work impairment trajectory class and the predicted class using the baseline characteristics. Figure A3. Individual trajectories of participants within activity impairment trajectory classes. Figure A4. Confusion matrix of the actual activity impairment trajectory class and the predicted class using the baseline characteristics. [file 12891_2023_6322_MOESM1_ESM.pdf]
